# *Being a patient in the intensive care unit*: a narrative approach to understanding patients’ experiences of being awake and on mechanical ventilation

**DOI:** 10.1080/17482631.2024.2322174

**Published:** 2024-03-03

**Authors:** Marte-Marie Wallander Karlsen, Lena Günterberg Heyn, Kristin Heggdal

**Affiliations:** a Department of postgraduate and master studies, Lovisenberg Diaconal University College, Oslo, Norway; b Department of postgraduate and master studies, University of South-Eastern Norway, Kongsberg, Norway; c Faculty of Health Sciences, VID Specialized University, Oslo, Norway

**Keywords:** Intensive care patient, narratives, narrative method, patient experiences, qualitative research

## Abstract

**Purpose:**

Intensive care patients often struggle to communicate due to the technical equipment used for mechanical ventilation and their critical illness. The aim of the study was to achieve a deeper understanding of how mechanically ventilated intensive care patients construct meaning in the unpredictable trajectory of critical illness.

**Methods:**

The study was a part of a larger study in which ten patients were video recorded while being in the intensive care. Five patients engaged in interviews about their experiences from the intensive care stay after being discharged and were offered the possibility to see themselves in the video recordings. A narrative, thematic analysis was applied to categorize the patients’ experiences from the intensive care.

**Results:**

A pattern of shared experiences among intensive care patients were identified. Three main themes capture the patient’s experiences: 1) perceiving the intensive care stay as a life-changing turning point, 2) being dependent on and cared for by others, and 3) living with negative and positive ICU experiences.

**Conclusion:**

The patients’ narratives revealed how being critically ill affected them, and how they understood their experiences in relation to themselves and their surroundings. The results can be used to pose important questions about our current clinical practice.

## Introduction

Imagine being critically ill, waking up with a tube in your mouth, and being connected to a lot of technical equipment in an unfamiliar room without your family present and without the ability to express your needs and concerns. This is the case for numerous patients in intensive care units (ICUs) around the world. The benefits of using fewer sedatives and analgesics on critically ill patients have been studied thoroughly from a medical perspective, and reductions in the mortality rate and the time needed for mechanical ventilation weaning as well as shorter ICU and hospital stays have been reported (Devlin et al., 2018; Schweickert & Kress, 2008; Strøm et al., 2010). Due to the guidelines promoting lighter sedation, ICU professionals are now caring for more conscious and alert patients during mechanical ventilation, resulting in the development of a more individualized and patient-focused plan for the use of sedation and analgesia (Devlin et al., 2018; Olsen et al., 2020; Strøm et al., 2010; Vincent et al., 2016).

Accoring to a Nordic meta synthesis of 15 qualitative studies about patients experiences in the ICU (Egerod et al., 2015), patients who have been interviewed retrospectively about their ICU experiences have expressed a feeling of being in limbo. Dependence of the health care professionals and struggle with communication because of critical illness and ventilator treatment has been highlighted as especially important experiences in several studies (Baumgarten & Poulsen, 2015; Carroll, 2007; Happ, 2000; Karlsson & Forsberg, 2008; Laerkner et al., 2017). Especially the complex communication barrier affect the relationship with the professionals and has consequences for the care provided (Karlsen et al., 2022; Laerkner et al., 2015; Tingsvik et al., 2013).

Lindberg et al. (2015) interviewed former ICU patients to obtain more knowledge about their experiences of autonomy in intensive care. Their findings revealed that patient autonomy could be seen as a trajectory towards partnership as the patients felt dependent having to surrender to the professionals’ control, but also were invited to participate in their care. Being able to participate created a trusting and healthy care environment (Lindberg et al., 2015). Danielis et al. (2020) did a meta-analysis of nine qualitative studies and found that the patient’s experiences while being mechanically ventilated in the ICU could be categorized into four themes: The perceived support from the family and loved ones, the effect of the intense stress on body´s system, the induced negative emotional situations, and the feeling of being cared for in a hospital setting. Experiencing uncomfortable symptoms such as pain, thirst, and breathlessness has also been an important finding from previous studies about the patients’ experiences (Alexandersen, Haugdahl, Paulsby, et al., 2021; Berntzen et al., 2018; Puntillo et al., 2010).

Many former intensive care patients (ICU patients) struggle with health problems long-term as many develop post-intensive care syndrome (PICS) (Brown et al., 2019; McPeake & Mikkelsen, 2018; Nanwani-Nanwani et al., 2022). The meaning that patients attribute to their life experiences influences how they cope with their experiences in the ICU. The patient’s cognitive, psychological, and physical functions can be affected after an ICU stay, resulting in complex care needs after the intensive care stay (Schofield-Robinson et al., 2018). Many patients also experience delirium during their ICU stay (Krewulak et al., 2018). Memories of ICU stays may be distorted by scary hallucinations and difficulties related to understanding the unfamiliar environment, which can make it hard to cope with the experiences from their ICU stay (Egerod et al., 2015; Jones, 2013; Russell, 1999; Tolotti et al., 2018). It is evident that ICU stays influence patients in many ways, including their sense of coherence, quality of life, and daily functioning (Schofield-Robinson et al., 2018; Valsø, Rustøen, Skogstad, et al., 2020; Valsø, Rustøen, Småstuen, et al., 2020; Yuan et al., 2021).

Although the experiences of former ICU patients have been studied from various angles for decades, knowledge on this topic remains limited (Carruthers et al., 2018; Egerod et al., 2015; Laerkner et al., 2017). A challenge for retrospective studies is that patients often struggle to remember details about their stays, even if they are awake while on mechanical ventilation. Furthermore, the needs of long-term ICU patients who are conscious while on mechanical ventilation may differ from those of patients who stay in the ICU for only a day or two (Alexandersen et al., 2021). ICU patients can therefore not be classified as a homogenous group, as their illness trajectory can vary considerably. A patient trajectory often involves a sequence of singular events or turning points related to an illness. A trajectory can often be unpredictable, as it might be alternating between acute and stable phases and does not have a set course (Pescosolido, 2013).

It is important to continue studying these patients’ perspectives from different methodological angles in order to achieve a deeper understanding of their experiences. Narratives obtained from patients after discharge may offer valuable insights into how they cope with their ICU stay and can be used to reflect on our current practice. To the best of our knowledge, we have not found any studies in which patients were interviewed post-discharge after being video recorded during their ICU stay and being offered the possibility to watch the actual video recordings afterwards. Combining methods such as these might offer valuable insight.

The aim of the present study was to achieve a deeper understanding of how mechanically ventilated intensive care patients construct meaning in the unpredictable trajectory of critical illness.

## Methods

### Design

This study was part of a larger study in which 10 patients who were awake while on mechanical ventilation were video recorded during their ICU stays, during 2016–2017. After they were discharged, the patients were invited to engage in individual interviews about their ICU experiences, and a narrative, thematic approach was used to analyse their stories (Riesmann, 2007). The study is reported in accordance with the SRQR Checklist(Appendix1).

### Participants and data collection

We recruited patients (*n* = 10) from two level III ICUs in the same University Hospital in South-Eastern Norway who agreed to be video recorded while in the ICU. The recruitment procedure and consent process were conducted in the following way: 1) Patients were approached by a trained department nurse who asked if they were willing to be visited by a researcher if they were eligible for the study. Inclusion criteria were that they were mechanically ventilated (for at least 48 hours), conscious and alert (with a Richmond Agitation-Sedation Scale 0–2) (Sessler et al., 2002), and not scored positive on delirium using the Confusion Assessment Method (CAM-ICU) the last 24 hours or appear in other ways delirious (Ely et al., 2001). Patients who did not speak Norwegian or had severely impacted visual, hearing or cognitive capabilities or were in end-of-life care were excluded. 2) Patients who volunteered, received a visit from the first author who explained more about the study and achieved non-vocal consent by nodding writing, or forming yes with their lips to engage in the video recording and to engage in interviews post-discharge. 3) The patients’ relatives were informed about the study and the videorecording was planned for the next day. 4) Patients received additional information and confirmed their participation with written consent once they were extubated and left the ICU, and 5) A time point for the interview was planned with the first author, a critical care nurse with over ten years experience from intensive care.

Two of the patients died before being discharged from the hospital, and one patient was discharged to palliative care and died shortly afterwards. We did not manage to get in contact with two of the patients after the ICU discharge despite calling and sending written invitation letters. The remaining five patients – two women and three men aged 43–72 years—all agreed to be interviewed. The recruitment process for the video recordings took 14 months in total (April 2016-May 2017). After a year the recruitment process stopped due to practical reasons, but also because the video recordings were proven to be extensive containing a rich variety of interactional data material. Results from the analysis of video recordings have been reported elsewhere (Karlsen et al., 2019, 2020). Table I describes the patient demographics of the five patients who engaged in interviews.

**Table I.** Patient demographics.

Age	Gender	Days on mechanical ventilation*	Place for interview	Duration interview (minutes)	Watched segments of video recordings
Median: 60 (range 43–72)	3 male 4 female	Mean: 21 (Range 15–30)	3 hospital 2 home	Median: 45 (Range 18–104)	Yes: 3 No: 2

*This refers to the days on mechanical ventilation at the time of the video recording.

The study participants were admitted to intensive care for both medical and surgical reasons, and the mean ICU stay before the video recordings was 21 days. In comparison, the average ICU stay in the hospital where we included the patients, was respectively 4.1 days and 2.5 days the year of inclusion. The ratio of nurses was 1:1, and in procedures such as mobilization or morning bath, they received assistance from other nurses. All the patients were on a ventilator, and most of them were in a weaning phase. This meant that they during the video recordings had time off the ventilator, trying either to use a speech-valve or a nasal cannula for shorter periods. Other procedures occurring regularly during the video recordings were visits from physicians or radiologists for a pulmonary X-ray. The ICUs had implemented the principles of analgo-sedation years before this study with the goal to reduce the sedation and focus on analgesia as well as implementing systematic scoring of pain, sedation level, and delirium. The ICUs had an open visitor policy, allowing frequent visits from their families. The ICUs did not have a routine for writing patients’ diaries or offering follow-up services after the ICU stay.

The participants were interviewed by the first author, and this ranged from one month and ten days to seven months and eleven days after their discharge from the ICU. Three of the five patients were interviewed within the two first months after discharge. Two participants were interviewed at home, and three patients were interviewed at the hospital. The reason for the variety of times to the interview after the ICU stay depended on the patient’s conditions, and how quickly they felt ready to be interviewed about their experiences. Giving the patients the possibility to choose the location for the interview allowed them to choose what was most comfortable for them. The patients were asked how they experienced being a patient in the ICU as well as what they remembered from their stay. The interviewer invited the patients to express themselves freely and to tell their stories in their own words. The interviews were recorded on a digital audio recorder. Furthermore, the patients were invited to watch and comment on video recording segments from their ICU stay in order to facilitate their memories.

### Analysis

The interviews were audio-recorded and transcribed verbatim by the first author. The analysis framework was inspired by Riesmann’s (2007) narrative approach to thematic analysis.

Three of the participants were willing to watch some of the video recordings once the interview had started and they told the researcher their initial reflections about their ICU stay. Watching selected video segments gave the patients the possibility to describe their experiences and get some new insight or stimulate their memory further. The selected segments could be of an interaction with a nurse or a physician and were not pre-chosen unless the interviewer had specific questions about a particular incident.

Each interview recording was listened to several times and the interview transcripts were studied thoroughly in order to familiarize with the text. The interview details were then condensed into a narrative with a focus on the stories of the participants, the words they used to express themselves, the way in which the events were described, and the individuals involved. Although many patients described the same phenomena, such as hallucinating and waking up on a ventilator, each story was unique. The analytic texts were read and commented upon by all authors in several meetings.

Story segments were initially analysed to capture the core of each patient’s experience. Then, they were analysed in a broader context to study shared patterns of experiences across patients’ (Nasheeda et al., 2019). Table II presents an example of the analysis of several patients’ experiences with uncomfortable symptoms. All the names in the presentation of the results are fictitous, to ensure patients anonymity.

**Table II.** Comparative analysis of patients’ experiences with uncomfortable symptoms.

*Patients*	*Ragnar*	*Anders*	*Lisa*
*Plot summary*	Living with uncomfortable symptoms	Living with uncomfortable symptoms	Living with uncomfortable symptoms
*Introduction*	Experiences of multiple bothersome symptoms, especially headaches, during the ICU stay	Experiences of uncomfortable symptoms, such as nausea and discomfort in the throat, during the ICU stay	Experiences of uncomfortable symptoms due to treatment in the ICU
*Problem/Conflict*	Normal daily procedures were painful.	Sometimes, the symptoms were so uncomfortable that the patient threw up and could not endure being awake.	The enteral tube hurt, and the patient wanted it removed.
*Climax/action*	Relatives said that what was being done to the patient looked uncomfortable and that he never had migraines or headaches before that time.	The intensivist decided to sedate the patient and said good night.	The patient received sleeping medication so that she could endure the discomfort and relax during the night.
*Resolution*	Being acknowledged and seen in uncomfortable situations	Sedation and analgesia when the discomfort was too much to endure	Sedation and analgesia
*Characters/time/setting*	The patient, health care professionals, and relatives in different settings	The patient and a female intensivist	The patient; health care professionals; and the patient’s husband, who witnessed her discomfort
*Coda*	Suffering during the ICU stay	Bothersome symptoms	Suffering during the ICU stay

**Figure 1.**
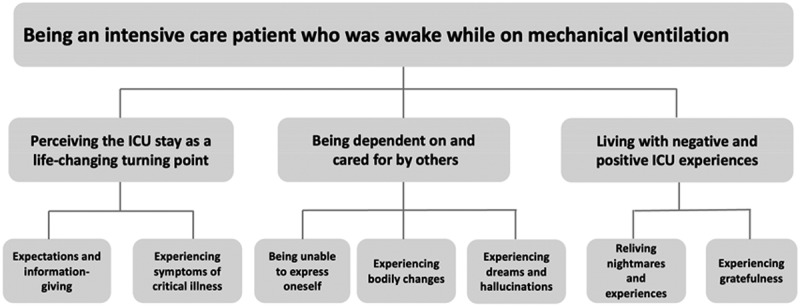
Themes and subthemes.

The next analysis step encompassed the categorization of the themes to identify subthemes as illustrated in Figure 1. The quotes in the presentation of the results were translated from Norwegian to English for this article.

### Ethical considerations

The study was approved by the South-Eastern Regional Committee for Medical and Health Research Ethics in Oslo, Norway (2015/2012). Furthermore, it was carried out in accordance with the Code of Ethics of the Helsinki Declaration (World Medical Association, 2013). The interview details, including digital files, transcripts, and analysis, were securely stored on a digital server in accordance with the university hospital’s regulations. Each participant signed a written consent form and received verbal information about the study as described in the method section. They were also made aware they could withdraw from the study at any point without any consequences. The interviewer was careful when asking about the participants’ ICU experiences. In addition, it was explicitly expressed that watching the video recordings was voluntary. Two of the five interviewees did not want to see the video recordings.

## Results

Three main themes captured the participants experience of being an intensive care patient who was awake while on mechanical ventilation, from the moment of becoming critically ill until the time they were interviewed. The themes were as follows: 1) perceiving the ICU stay as a life-changing turning point, 2) being dependent on and cared for by others, and 3) living with negative and positive ICU experiences. Themes and subthemes are presented in Figure 1.

### Perceiving the ICU stay as a life-changing turning point

#### Experiencing symptoms of critical illness

In some cases, the critical illness that led to the ICU admission of the participants had been present for some time. Most participants felt that something unpleasant and serious had happened to them before they were admitted, and they experienced uncomfortable symptoms, such as pain. The story of becoming critically ill was unique for each participant; however, the joint experience was often an acute, critical incident that led to hospitalization. This circumstance is evident in the following interview excerpt:

I was in the hospital in May. I was horribly exhausted, but they took a chance on operating me because they thought I could handle it. And I just had to accept it because I had blood clots and a new tumor, which grew towards the heart, and my veins were filled with shit.

Patient Elisabeth

#### Expectations and information-giving

The admission to the ICU was experienced as dramatic. One interviewee described how he had 20 minutes to prepare for surgery while simultaneously making sure that someone took care of his cats and threw away his trash while he was in the hospital. This was not an expected course of action when he first decided to visit the Emergency Unit to assess the pain he experienced in his jaw after a dentist appointment. He was rushed to the hospital, where he was prepared for surgery and told that he would stay in the ICU for a couple of days. The next thing he remembered was waking up several weeks later.

I understood what they had done, but not how close it was to end badly … because I think that must have been the case many times during the stay.

Patient Oscar

While reflecting upon the ICU stay, another patient; Ragnar, expressed how he felt looking on himself at the video recording in the following way:

I was not much of a man there . I didn´t think I was that helpless

Lisa also reflected upon her ICU stay as she said the following before she became quiet for a while:

It is very difficult to think that I have gone through that and survived

#### Being dependent on and cared for by others

Waking up on a ventilator was described as a strange, unexpected feeling. Concerns included a sense that time had passed, a desire to know what had happened, and a feeling of being affected by medication. As patients tried to understand what they described as a vacuum, they simultaneously felt grateful that the professionals had saved their lives and tried to make sense of their environment and what was happening around them. Patient Ragnar went through the following thought process:

I remembered that I saw that it was the 12^th^ or 13^th^ of June and [my first thought was] what on earth is going on? I had surgery [on] the 25^th^ of May, and it was expected that I should be out [of the hospital on] the 27^th^ or 28^th^. I did not understand anything.

The patients’ stories revealed that life in intensive care was not easy. At the same time, they found it comforting that they did not need much besides support from the professionals, who continuously observed, cared for, and attended to them. This created a dual feeling of being treated like a child who is dependent on others and wanting to be more independent and do things on one’s own. Elisabeth said that she felt like a little child who needed help with everything and who tried to trust the people around her. Meanwhile, Ragnar said that it seemed like time just “boiled away” because he was okay and felt safe in the presence of the professionals and his family. Later in the interview, a statement revealed how he felt about a certain nurse whom he observed in the video recording:

It is just the fact that she comes and holds my hand, you feel such care. and security by that gesture. I remember letting go of her hand there in the video because it got too warm, but she came over like that many times and it was so friendly. Many such small episodes are coming back to me now.

#### Being unable to express oneself

After waking up on a ventilator, the patients quickly realized that they were unable to communicate normally. They described how they struggled to express themselves and establish connections with ICU personnel. This challenge led to feelings of being bound to and dependent on the devices and professionals around them. The communication problems made it difficult for patients to sustain relations with their families and establish social relations with professionals. Patients’ stories contained positive examples in which being assisted with communication was a good experience and negative examples in which unsuccessful communication created inner frustration. Participants described wondering if their voices would ever return to normal. Lisa said:

I could not speak or move, and then I was supposed to push these letters [on a communication board], but I couldn’t do that either … because I felt so heavy in my arms.”

Some patients reflected on their interpretation of the situation of being non-vocal. One participant shared the following experience:

I thought, how can they not understand me? It was very easy for me to think, how stupid can they be . They must understand what I mean, right? That was my thought, but now I understand that if someone would talk to me just [by] moving their lips, I would not have understood what they meant either. So, I really should have thought the opposite.

Patient Ragnar

#### Experiencing bodily changes

The patients described how their bodies changed during their critical illness. They noticed these changes in many situations, such as during mobilization as well as while observing themselves and their wounds, seeing that they were connected to technical equipment, and realizing that their bodies did not function normally. The feeling of a heavy, uncomfortable, weak, and changed body was interpreted as a recognition of the extent of the critical illness. It also meant that the patients had to adjust to their bodies, as they suddenly realized that they could not do everything that they wanted and that recovering would take time. According to Oscar, “*I could not even move my hands or use them. They were heavy as lead, and I could not move, I could not even lift my arms.”*

The patients developed motivation, willpower, and strength by fighting to endure heavy training and pushing themselves to try to do more than they thought they could without quitting. The patients described how the professionals often encouraged them to take even small steps in their recovery. Even a normally easy task such as breathing became hard, and something they became very aware of. Elisabeth had the following reflections as she watched the video recording from the stay when the physicians and the nurse were talking to her about weaning off the ventilator:

I remember that it was. I had such a hard time breathing I barely dared to sleep because I felt I had to watch my breath. So I had to lay there and look after myself breathing and that was exhausting.

Patient Elisabeth

Feeling uncomfortable was described as an inevitable part of the ICU stay. These sensations could be physical, such as pain or vomiting, or psychological, such as fear, anxiety, or hallucinations. Even routine procedures could be uncomfortable to the extent that it was impossible to endure being awake. Being relieved of pain through sedation and analgesia was a positive experience for the patients. These uncomfortable symptoms were understood as a dilemma, and several patients described how they pushed themselves through feelings of nausea or vomiting just to be able to express themselves for a short period of time while using a speech cannula. Family members and professionals often witnessed this suffering, which had an essential impact on social interactions as they had problems understanding the patients’ expressions.

#### Experiencing dreams and hallucinations

The patients’ stories contained thoughts, hallucinations, and dreams about things that were not real. However, they felt real in the moment and often revolved around death, travelling places, or being kidnapped. One participant shared the following experience:

I dreamt that I went into an elevator that drove up and down … beside me there were these small windows where faces popped up and controlled me … When I looked to be completely exhausted, they pinched a little needle in me and looked for my feelings and then they said, “Now you are dead” I think I heard around two [or] three times [that] I was dead, but I thought it was not so bad … because it was so exhausting to drive that elevator up and down.

Patient Elisabeth

Another participant shared the following experience:

I was kidnapped and taken to Thailand and drugged … They were [going] to give me AIDS. I saw those famous mountains, and I was sure I was there, but then my husband said, “You are at the hospital.”

Patient Lisa

Living with a distorted reality became a part of patients’ lives while in intensive care. It was often challenging for the patients to describe these experiences to the professionals because of communication barriers. Feelings of anxiety and fear were viewed as normal reactions to the hallucinations, and professionals and family members fostered a feeling of safety by reassuring the patients that nothing bad would happen to them.

### Living with negative and positive ICU experiences

#### Reliving nightmares and experiences

Being in intensive care marked the patients physically and psychologically. This was evident when patients showed the interviewer their scars and talked about numbness in the body areas where they had surgery. In addition, patients told the interviewer that they were still struggling with thoughts about their ICU experiences, including the hallucinations. Patients felt alone with their thoughts and imagination. Although they were offered follow-up services from their general practitioner, these visits mainly addressed physical problems; their psychological reactions and existential struggles were not necessarily captured in any health care encounters. According to Lisa, *“It is very weird what I have been through, very weird [… and] almost unbelievable.”*

Families’ stories from intensive care became a part of the patients’ memories. Some patients experienced gaps in time as well as in their memories of episodes that had been described to them. Therefore, they had to adapt to not remembering parts of their ICU stays. Anders shared the following experience: *“The kids have told me some stuff so I kind of know this and that but I do not go around and think about it.”*

Negative emotions, depression, feeling flat, and crying were not unnormal and were seen as a reminder of frailty. The participants expressed a feeling of loneliness and of being the only one who understood their experiences. Having been through it all on their own was an emotional burden they had to carry after discharge. One participant shared the following experience:

I think something has happened to me after that surgery without being able to pinpoint what. Everything is so heavy, and it is most comfortable for me just to be calm, but it is not how it is supposed to be. [For] a couple of days now, [including] yesterday and the other day, she [the wife] has been complaining about me just sitting inside, but it is necessary to understand why I do that.

Patient Ragnar

Although some patients talked about the changes in their lives, not all of them wanted to be directly reminded of their ICU stays. On the contrary, they tried to distance themselves from this experience. For example, Oscar said, “*I don’t think about it that much [any]more . It is a chapter that is over, really, so it has been pushed back in the head, or . yeah, I am done with it.”*

The narratives indicated that some patients wanted to deny or suppress their ICU experiences because they were associated with so many negative emotions. However, many patients admitted that they frequently thought about their ICU stays. During the interviews, some participants had what were interpreted as expressions of physical defence mechanisms, which included projecting protective body language, being tense, tearing up, or becoming silent while reflecting on how their ICU experiences impacted their lives.

## Discussion

One of the main results from this study were that the participants clearly narrated the experience of becoming an intensive care patient as something dramatic and life changing. The narratives reveal how intense and detailed some patients’ memories were, even after considerable time had passed since their discharge from intensive care. The same observation has been made in other studies (Egerod et al., 2015; Russell, 1999; Tolotti et al., 2018). The participants frequently experienced hallucinations and had frightening memories of unreal experiences. For example, some patients thought they were being kidnapped and that the professionals could not be trusted because they were involved in the kidnapping. It might have been extremely difficult for the patients to talk about how it felt to be kidnapped and express how they felt in the situation – as they later realized it was not real. Moreover, the emotional, cognitive, and physical impacts of an ICU stay may affect the patient’s health for a long time after discharge (Alexandersen, Haugdahl, Paulsby, et al., 2021; Alexandersen, Haugdahl, Stjern, et al., 2021; Morgan, 2021). Our study reveals that patients still experience troublesome memories after the implementation of analgosedative practice and strengthens the belief that this continues to be an important part of the ICU experience.

The concepts of comprehensibility, manageability, and meaningfulness have been described as important aspects of an individual’s sense of coherence and health that help them maintain a certain degree of control over stressful situations (Antonovsky, 1987; Eriksson & Lindström, 2006). The interviews in the present study revealed that the patients struggled to understand their situation and communicate and interact with professionals. Furthermore, they were unable to influence their situation because the ventilation tube made it impossible to speak. The findings emphasize the experience of vulnerability of being critically ill and having to put one’s life in the hands of health care professionals.

As illustrated by the patients’ narratives, the new intensive care paradigm’s (Devlin et al., 2018; Vincent et al., 2016) emphasis on awake, active, and collaborative patients as the ideal has consequences for both patients and professionals. Many patients expressed wonder at what was happening to them and needed continual information to feel safe. This is demanding both for the professionals and the patient (Carruthers et al., 2018; Holm et al., 2021). The need for more interaction with patients and attention to their psychological needs should not only be addressed after the ICU stay; measures should already be in place when patients are on mechanical ventilation and conscious enough to understand their surroundings. Our study elicits how demanding and frustrating it is for patients to communicate while being on a ventilator. This is in line with previous studies (Carroll, 2007; Danielis et al., 2020; Holm et al., 2021). It might be justified to ask if it is beneficial for all ICU-patients psychological health and recovery to be awake and conscious as much as possible while being mechanically ventilated. This is an ethical question the professionals need to reflect upon, balancing the principles of beneficence and non-maleficence, while trying to embrace the new treatment recommendations in clinical practice (Kvande et al., 2021; Vincent et al., 2016). After this study was performed, a systematic review and analysis performed by Aitken and colleagues (Aitken et al., 2021) has investigated the effect of deep sedation on intensive care mortality, duration of ventilation and other clinically relevant outcomes. They have found that the evidence of benefits from lighter sedation is limited and inconsistent. The recommendation from Aitken et al. (2021) study is therefore to build evidence related to optimizing patient outcomes, both within and beyond the intensive care stay.

Several studies about patient involvement in the ICU have revealed (Karlsen et al., 2020; Laerkner et al., 2017; Lindberg et al., 2015), that the patient’s trajectory of involvement not only depends on their wishes, but on how the professionals manage to include and inform patients in the decisions. This study offers good examples of how the caring attitude of the professionals made the patients feel safe, and how this aspect of nursing is connected to the quality of communication and interaction with the patient. However, some of the patients expressed that they felt responsible for looking after themselves. This happened especially when struggling with the mechanical ventilation or the weaning phase, trying to watch their own breath. Episodes in which patients perceive a lack of care can make them feel lonely, afraid, and unacknowledged, while situations in which patients receive care could lead to opposite feelings (e.g., acknowledgement, security). By communicating and interacting with patients, professionals can create an atmosphere in which the patients feel safe and valued. This was confirmed in our study when the patients expressed how they felt taken care of and being watched over (e.g., patient Ragnar’s example of the nurse holding his hand and safeguarding him). Professionals and patients together can impact the way in which patients are included in their own care on a daily basis in the ICU (Karlsen et al., 2020). There is no standard of care regarding how much or little patient participation is appropriate. The complex patient situation in an ICU requires continual reflection on the part of the professionals in order to facilitate and individualize care and actively involve the patients.

### Creating a space for patients’ experiences

According to Antonovsky’s (1987) salutogentic principles, experiences involving stressors and tension are not always negative. If the expectations and demands of a situation are appropriate, experiencing stress and tension may even be seen as a coping strategy. However, the patients’ narratives in the present study indicated that the stressors and tension of the long-term ICU stays exceeded their ability to cope as they did not understand what was happening, had difficulties expressing themselves, and experienced many physical problems. In other words, they struggled to manage and understand the situation in a meaningful way. In a study of salutogenetic interventions, Langeland et al. (2022) stated that coping requires patients to actively adapt to or be involved in their situation, understand their own resources, and be supported in using them. In this context, professionals can play a vital role in strengthening patients by continually sharing information about what is happening during the ICU stay, encourage communication, and motivate patients to re-engage in activities of daily living. King et al. (2019) conducted a scoping review on needs for social support among intensive care survivors. Addressing the importance of creating adequate support systems from the period of ICU admission to discharge and adaptation to home/community care, King et al’s study argue the importance of taking into account the patients transition from the critical illness event, to the stabilization, preparation for discharge, and later adaptation to the new life at home. We recommend that such systems for social support is developed and systematized in order to promote health and recovery long-term.

Professionals might take for granted much of the patients experiences, as they are a part of the ICU-environment and more familiar with this context. Previous studies have revealed that professionals both misinterpret, struggle to identify symptoms sufficiently (Happ, 2000; Happ et al., 2011; Karlsen et al., 2019), and feel torn between their own ideals of wanting to deliver high quality care and the reality they face caring for a conscious and alert patient on mechanical ventilation (Karlsen et al., 2022; Laerkner et al., 2015; Tingsvik et al., 2013). Future studies should investigate and combine the experiences from the professionals and the patients, to further develop knowledge about the interaction.

The patients in this study did get the possibility to watch the video recordings from the ICU stay. The impact of this on the patient’s sense of coherence and coping skills has not been thoroughly explored but should be studied more in depth in the future. The patient interviews offered insight into how they looked back on the experiences from the ICU, and the fact that they had both positive and negative recollections from that point of time, and how strongly it had influenced their abilities to cope adequately. Karlsson and Forsberg (2008) found that patients mastered their lives after their ICU discharge by rewriting reality to reflect their current situation, tending to their soul as part of self-care, either avoiding or confronting memories of their ICU stay, and crying. The path towards healing may include being given detailed explanations of the intensive care environment, meetings with the professionals who cared for them during their ICU stay, engaging in a dialogue with professionals about their ICU experiences, and acknowledging that this type of life event causes permanent changes.

Patients’ narratives can help to guide clinical practice at the individual and organizational level. In our study it became apparent that the patients experienced struggles to communicate their needs. Individually, professionals have professional, legal, and ethical responsibilities related to understanding patients’ experiences and creating a safe, communicative environment. However, the health care services must provide sufficient resources to arrange a follow up with the qualities that the patients need, especially when it comes to communication, we know that many of the patients struggle with this while being mechanically ventilated. Furthermore, previous research has shown that one of the barriers to good communication in the ICU is the time pressure put on the professionals when trying to find out what the patients wonder about, whilst managing other tasks such as medication, mobilization, and other nursing procedures (Holm & Dreyer, 2017; Holm et al., 2021; Ijssennagger et al., 2018; Karlsen et al., 2022). The care and attention the patients receive during the ICU stay will affect their experiences.

The participants in this study did not receive any special follow-up services; however, some reported talking to general practitioners, friends, and/or family members. The patients described feeling flat or emotionally numb, which is an important finding not thoroughly described in previous studies. In an emotional context, flatness often means being without energy, without a sense of direction or engagement in ones life situation. Flatness does not necessarily mean that the person is neutral as it can be an expression of frustration or a way of distancing oneself from reality. Replacing bad feelings with happy feelings may evoke meaningful experiences and lead to a transformation, as “only feelings can change emotions” Barrett (2018), Greenberg (2018). Therefore, reconnecting with emotions, even painful ones, may reduce flatness. Paying attention to and focusing on the specific expressions of a patient’s emotions may be an essential method for reconnecting with their previous life and working through the impact of their ICU stay (Carruthers et al., 2018; Karlsson & Forsberg, 2008). Exploring the emotions impact in the follow-up services would be an interesting area for further development in terms of clinical practice and research.

The study findings indicated a need to look more closely at the type of environment experienced by ICU patients. Furthermore, the in-depth analysis of the patients’ narratives revealed the importance of establishing a humanizing ICU environment in which inner healing is part of the rehabilitation process towards a functional post-ICU life. A recent scoping review defined humanizing care as “holistic care of the patient,” “a general attitude of professionals toward the patients,” and “an organizational trait toward all subjects of the healthcare system” (Kvande et al., 2021, pp. 503–504). Interestingly, the participants in our study stated that the environment could be caring and humanizing as well as uncaring and dehumanizing. Karlsson and Forsberg (2008) described this dual experience of care and how it could impact the patients experiences. The feeling of a warm, close human connection with the professionals were in Karlsson and Forsberg (2008) study described as of crucial importance for the patients’ experiences and aligns with our study. The link between communication and interaction in the ICU and the effect on patients’ post-ICU recovery has not yet been explored, and further studies are warranted.

Based on our current knowledge (previous studies) about the impact of ICU stays on patients and this study’s findings, a life-altering event, such as ICU admission, should lead to systematic follow-up services that attend to physical, psychological, and existential dimensions (Valsø, Rustøen, Skogstad, et al., 2020; Valsø, Rustøen, Småstuen, et al., 2020). Our findings reveal that patients’ struggles involved unattended physical, mental, existential, and social needs that must be addressed. Follow-up care should be conducted within a multi-disciplinary team work approach, combining expertise from all the professionals in the ICU such as intensivists, physiotherapists, speech-language pathologists, and critical care nurses. Alexandersen, Haugdahl, Paulsby, et al. (2021) interviewed long-term ICU patients on mechanical ventilation after they were discharged. The findings revealed that professionals and relatives can contribute to the establishment of a health-promoting environment that reduces patients’ feelings of loneliness and worries about the future and builds their inner strength and determination to keep fighting. However, it can be hypothesized that a longer ICU stay has a greater impact, both in positive and negative ways, on patients’ relationships with professionals and family. This important topic has not yet been investigated and should be addressed in future research.

### Strengths and limitations of the study

The study’s small sample population allowed for in-depth reflection and a thorough analysis of the interview data. The retrospective recollections of the patients’ ICU stays happened at various time points and locations, which might have been a weakness when comparing patient narratives to identify themes. However, it may also have been a strength because it enabled the highlighting of various ICU experiences that the patients found important. It is also worth mentioning that we are always in continuous change as to how we understand our own experiences. This study is based on the participant’s accounts of their experiences at the time of the interview, and “create the very events they reflect upon” (Riesmann, 2007, pp. Chapter 7, page 3).

Although an inclusion criterion on length of stay was not developed for the study, each participant had an extensive ICU stay. Patients receiving long-term intensive care may have memories of several episodes and detailed recollections of building relationships with professionals over time, resulting in more thorough accounts of the ICU stay. We found that the data had rich information power (Malterud et al., 2016), as we had invited key persons to share their experiences about a very specific time in their life. An open dialogue was achieved during the interviews, helped by the contact the interviewer had with the participants while they were video recorded during their ICU stay on mechanical ventilation, up until the time of the interview.

Repeated interviews with the participants could have uncovered more relevant details and given the interviewer an opportunity to ask about points that were unclear or not touched upon in the first interview (Nasheeda et al., 2019). Repeated interviews were not planned in the main study, and the patients were only asked to participate in one interview. In addition, there was considered an ethical challenge conducting repeated interviews with patients who have had strong emotional experiences without a psychologist ready for follow-up. Furthermore, since the coronavirus disease 2019 (COVID-19) pandemic gave restricted access to former ICU patients, we decided to stick with the one-interview strategy.

### The study’s impact on clinical practice and education

The present study provides valuable insights that can inspire, serve to pose relevant questions and create ideas for improvement of clinical practice and education. Nurses need to consider the vulnerability of critically ill patients so that they can interact with them appropriately, empower them, and support their recovery. Furthermore, patients need a humanizing environment, which professionals can create in several ways, such as through communication and interactions with patients and their families.

After the interviews were conducted, an increased awareness regarding follow-up services for ICU survivors in several countries has occurred, including Norway, the United Kingdom, and the United Stated of America. However, the health-related outcomes of ICU follow-up services have up until this date not been sufficiently investigated. Moreover, the follow-up services provided to date have varied in terms of length, contributions from various professionals, and focus (e.g., physical rehabilitation, psychological support) (Schofield-Robinson et al., 2018). Meanwhile, the COVID-19 pandemic has strengthened the focus on the physical rehabilitation in follow up-services of ICU survivors because persistent respiratory problems, fatigue, myalgia, and cognitive deficits are potential obstacles to healthy psychological rehabilitation (Amdal et al., 2021).

There is a need for more research to identify best practices in working with patients experiences of the ICU; ways to overcome substantial hurdles, such as economic and physical barriers (e.g., funding, location); proper screening tools to identify patients at risk of PICS; and which professional groups should collaborate on the development and implementation of follow-up services (Butcher et al., 2022; Jones, 2013; Valsø, Rustøen, Skogstad, et al., 2020; Valsø, Rustøen, Småstuen, et al., 2020).

Educational training for ICU should focus on how communication and interaction can affect patients’ long-term health and quality of life. Nurses specializing in intensive care may have an effect of training programmes focused on advanced communication skills and the use of simulation-based techniques (Karlsen et al., 2021). Training programmes should also prepare health care to provide high-quality, evidence-based follow-up services for former ICU patients.

## Conclusion

The present study revealed three main themes that captured experiences of being a critically ill patient on mechanical ventilation in an ICU: 1) perceiving the intensive care stay as a life-changing turning point, 2) being dependent on and cared for by others, and 3) living with negative and positive ICU experiences. The patients’ narratives showed that being critically ill has deep physical, cognitive, emotional, existential, and social effects on the person involved. The present study provides valuable insights that can inspire, serve to pose relevant questions, and create ideas for improvement of clinical practice.

## Supplementary Material

Biography.docx

SRQR_Checklist.docx
