# Supplementary material for: Being a patient in the intensive care unit: a narrative approach to understanding patients’ experiences of being awake and on mechanical ventilation
Source: Int J Qual Stud Health Well-being. 2024 Mar 3;19(1):2322174. doi: 10.1080/17482631.2024.2322174 (PMC10911109; doi:10.1080/17482631.2024.2322174)
Supplement: Biography.docx [file ZQHW_A_2322174_SM9568.docx]

**Biography**

*Marte-Marie Wallander Karlsen*

Karlsen is a critical care nurse, Associate Professor and Program Director for master studies at Lovisenberg Diaconal University College, Norway. Her PhD focused on communication with mechanically ventilated patients in the intensive care unit, where she video-recorded interactions between patients and providers. Dr Karlsen’s research field also focuses on how to advance nurses communication skills, using standardized patients in simulation-based settings as well as how unexperienced registered nurses experience the transition into working in intensive care units.

*Lena Günterberg Heyn*

Heyn is a Professor at the University of South-Eastern Norway, where she is leader for Center for Health and Technology and Research leader Strategic Initiative USN Health and welfare services of the future. Her research field involves communication in different settings, such as cancer care and intensive care, in addition to communication with technology. She also teaches and research communication skills training, and feedback in simulation (including virtual simulation). Heyn is an Associated Editor for PEC Innovation and has edited a book on communication in Norwegian.

Kristin Heggdal

Kristin Heggdal is a professor in Nursing at the Faculty of Health at VID Specialized University in Oslo, Norway. Dr. Heggdal has extensive experience in teaching and supervision students at the bachelor, master, and PhD level of nursing and health sciences. Her main research interests are directed towards patients` and families` resources for health while facing chronic illness. She has a strong interest in developing and testing of generic interventions that can be used across diagnostic categories and clinical settings to strengthen health within illness.
